# Commentary: Does Blue Uniform Color Enhance Winning Probability in Judo Contests?

**DOI:** 10.3389/fpsyg.2018.01213

**Published:** 2018-07-23

**Authors:** Víctor Cárdenes, Jorge C. Lafuente, Raúl Merinero, Álvaro Rubio-Ordoñez

**Affiliations:** ^1^Department of Geology, University of Oviedo, Oviedo, Spain; ^2^Departmento de Educación Física y Deportiva, Universidad de León, León, Spain; ^3^Department of Crystallography and Mineralogy, Complutense University of Madrid, Madrid, Spain

**Keywords:** judo, color, bias, competition, contest

The commented paper Dijkstra et al. (2018) is an exhaustive work which sheds light on the role of the white and blue judogi (judo outfit) in judo contests. The importance of colored uniforms has been proven in other sports (i.e., Attrill et al., 2008; Falcó et al., 2016; Krenn et al., 2017). During the recent 2017 World Cup races in Stavanger, Norway, several speedskating teams changed their traditional colors to different shades of blue, since it was believed that “blue is faster.” There is no scientific evidence to back up this statement, but the skaters, in fact, felt faster.

The use of the blue judogi was first suggested by A. Geesink in 1986 and introduced in 1997. This change was made to enhance the visualization of the contest. Prior to this change, the competitors were identified by wearing a red or white belt besides their own belt. With the introduction of the blue judogi, the competition became clearer and easier to understand for the general public. However, for some in the judo world, the blue judogi was contrary to the tradition and the spirit of judo (Matsumoto et al., 1997). In the following years, several papers were published pointing out the existence of a color-driven bias (Rowe et al., 2005; Matsumoto et al., 2007; Julio et al., 2015), and others showing no bias at all (Dijkstra and Preenen, 2008). There was no consensus about this question, which is a significant one for a sport that is practiced at all levels, up to and including the Olympics. The results of these studies were negatively affected by the small number of contests analyzed (400–1,600) and also by the seeding effect. Dijkstra et al. (2018) have solved this uncertainty by analyzing more than 42,000 contests, making their conclusions statistically representative. According to Dijkstra et al. (2018), there is a bias toward the “first called athlete,” who has a greater chance of winning the match, especially from the sixteenth (1/16) round. This “first called athlete” bias is independent of the judogi color. Before 2011, the athlete wearing the blue judogi was called first, and since 2011 the one wearing the white judogi has been called first. In both cases the bias favored the first called athlete. For Dijkstra et al. (2018) this bias would be due to factors like seeding, skill of the athletes, or even intercontest intervals. However, we have two remarks that are intended to complement the conclusions of Dijkstra et al. (2018).

## The “first called athlete” bias vs. “right hand of the referee” bias

The first called athlete is always at the right hand of the referee. The referee is the person who judges the athletes' actions, evaluating those actions according to International Judo Federation (IJF) rules without any nuance. Nevertheless, the referee's decisions might be influenced by subjective factors (Plessner and Haar, 2006) inherent to the contest, and also by his/her own motor experience (Dosseville et al., 2011). As is true of the general population, 90% of the referees are assumed to be right-handed (Scharoun and Bryden, 2014). The relative position of the athletes in relation to the referee might explain the bias toward his/her right hand. The referee might pay more attention to events happening to his/her right than to the left. Another interesting point is that, in most of the video contests (Olympic Games and World Championships, source: www.youtube.com) that were observed, the referee tends to move to his/her right side immediately after the start/restart of the contest. This fact might suggest that the referee has a better view of the athlete on the right and thus is prone to give him/her better scores. If this is the case, the “first called athlete” bias might be more accurately called the “right hand of the referee” bias.

## Asymmetry

**Figure 1:**
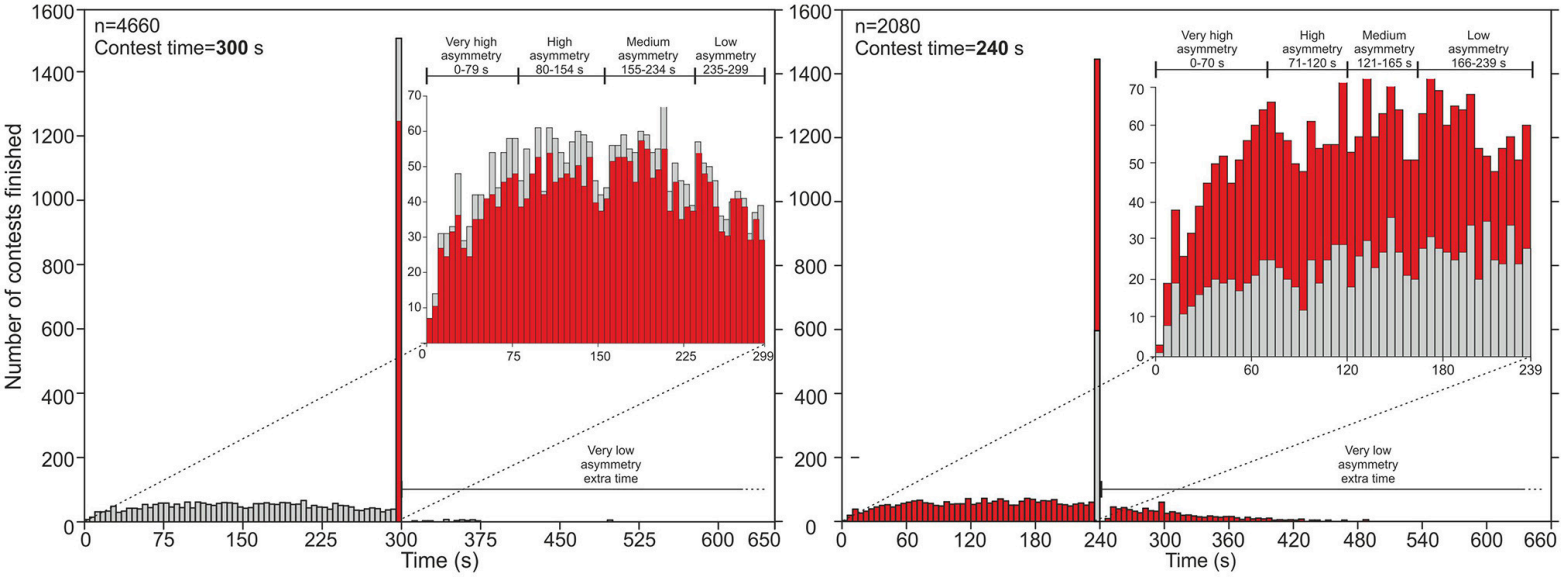
Number of contests finished (y axis) vs. time in seconds (x axis). Left graphic for contest time = 300 s, right graphic for contest time = 240 s. In gray, data for men; red for women's data. Data retrieved from judobase.ijf.org.

The asymmetry between athletes is the degree of dissimilarity between their fighting skills and is the decisive factor in who wins the match. Previous studies considered this asymmetry to be higher in the early rounds, due to seeding (Rowe et al., 2005; Dijkstra and Preenen, 2008; Julio et al., 2015; Dijkstra et al., 2018), and so the results of the early rounds have been thought to be biased by seeding. For us, the asymmetry can be measured by the contest's duration. When there is great asymmetry, the contest time will be short, since the winner will not have much trouble in ending the match. On the other hand, when both athletes have similar skills (low asymmetry), the contest will probably last the full duration. We analyzed the duration of 6,410 contests (Figure 1) from the same datasheets used by Dijkstra et al. (2018), finding four intervals of asymmetry, which roughly coincide with the quartiles of the contest time measured in seconds, plus a fifth interval of very low asymmetry, defined when the contest continues into extra time. We analyzed 300- and 240-second contests. Usually, 300-second contests are used for men's categories, while 240-second contests are used for women's categories, but this depends on the organization rules of each competition. Thus, asymmetry is defined by five categories: very high asymmetry (contest ends in the 1st quartile, Q1), high asymmetry (Q2), moderate asymmetry (Q3), low asymmetry (Q4), and very low asymmetry (extra time). It is notable that for both men and women the asymmetry intervals are the same. These asymmetry intervals describe accurately the intrinsic differences between the opponents.

## Concluding remarks

The commented paper (Dijkstra et al., 2018) is a complete and solid work that has shown that the color of the judogi does not produce a bias in judo contests, unlike in other combat sports in which the color red definitively causes a bias (Hagemann et al., 2008; Falcó et al., 2016). Instead, in judo there is another bias, not related to color, called the “first called athlete” bias. The reason for this bias is still uncertain, but we strongly believe that it is due to the referee and his/her dominant hand. This conjecture is interesting enough to merit further research.

## Author contributions

VC wrote the manuscript and analyzed the data. RM analyzed the data. JL and ÁR-O drew the graphics and wrote the manuscript.

### Conflict of interest statement

The authors declare that the research was conducted in the absence of any commercial or financial relationships that could be construed as a potential conflict of interest.
